# Socialization Practices Regarding Shame in Japanese Caregiver–Child Interactions

**DOI:** 10.3389/fpsyg.2019.01545

**Published:** 2019-07-11

**Authors:** Akira Takada

**Affiliations:** Graduate School of Asian and African Area Studies, Kyoto University, Kyoto, Japan

**Keywords:** caregiver–child interaction, Japanese, shame, language socialization, affect

## Abstract

Affect is both an organizing force and a product of socialization practices in communities. Shame is an affective experience that is primarily rooted in socially shared normativity, and it has featured in studies of language socialization that examine how children are socialized into their socio-culturally structured universe (Duranti et al., 2012). After the publication of Benedict’s (1946) seminal work, shame became associated with the ethos of East Asian cultures. Inspired by previous work, this paper focuses on the use, in socialization, of phrases that include the Japanese term *hazukashii*, which is commonly translated as shameful, in the context of Japanese caregiver–child interactions. We videotaped interactions between young Japanese children and their caregivers in natural settings and examined the gestures and speech around uses of *hazukashii*. The results indicate that phrases including *hazukashii* are often used when a child hesitates to perform an appropriate action or performs an act that is deemed inappropriate. The caregiver thereby provides an account that the action is understandable in the given context. Further, *hazukashii* is also used in teasing contexts. This is done to promote a cooperative and pleasant atmosphere. The word *hazukashii* is a powerful tool for the language socialization of children in Japanese speech communities.

## Introduction

Affect is both an organizing force and a product of socialization practices in various communities. It merits the fullest consideration: even if an emotion is commonly observed across various speech communities, the cultural meaning of that emotion in relation to the dominant values of the speech community could differ. Moreover, when emotional terms are used to describe an action or the state of a particular person in a conversation, whether or not it is the person him- or herself stating it, the emotion is not necessarily internally experienced by that person. Thus, Averill (1980, p. 337) posited that emotions are part of the socially constructed role that a person plays. He also asserted that it was necessary to analyze emotions on a socio-cultural level rather than on a physiological level. We must consider emotional expressions used in mundane, everyday interactions to understand emotions properly as socio-cultural constructs (Demuth, 2013).

Shame is among the affective experiences that Ekman (1992) listed as the basic emotions.^1^ It is primarily rooted in socially shared normativity, and it has attracted considerable attention in the study of social history and socialization in East Asian (e.g., Benedict, 1946; Doi, 1973, 1974; Clancy, 1986; Fung, 1999; Fung and Chen, 2001; Lo and Fung, 2012) and other (e.g., Schieffelin and Ochs, 1986; Fader, 2006; Reynolds, 2008; Demuth, 2013) societies. Below, I give a brief summary of important studies of socialization into and through shame in East Asian societies.^2^

Ruth Benedict’s *The Chrysanthemum and the Sword (1946)* was one of the most influential discussions of Japanese society published during the immediate postwar period. Benedict was leading the Japanese team of the War Information Bureau when she was conducting the research for this book, which was based on interviews with Nikkei, or Japanese emigrants living abroad; in Benedict’s case, these were Japanese who had emigrated to the United States and their descendants. While she was conducting her research, its subjects were living in wartime concentration camps. Benedict was a leading cultural anthropologist in an academic environment that adhered to the doctrine of cultural relativism. In her research, she sought a unique ethos expressed throughout Japanese culture. She wrote that the Japanese were extremely sensitive to the expectations and criticism of others (including their family members, stakeholders in their profession, and the general public) and that their social lives were strongly bound by ideas of grace and obligation. Benedict characterized Japanese culture as having a foundation in feelings of shame. Moreover, she approached the Japanese “culture of shame” through its contrast with the Western “culture of sin,” which, she proposed, could be understood as being based on the feeling of sin, which is present in each of its members through the enlightenment of conscience in reference to absolute moral standards. Many Japanese, including researchers, marveled that Benedict, who never visited Japan, was able to analyze Japan’s culture and the spiritual life of its people in such a beautiful writing style.^3^

The influence of Benedict’s works, including *The Chrysanthemum and the Sword*, is clear in much scholarly work and general discussion on Japan, even within the country itself. A notable example of this is the work of the Freudian psychiatrist and theorist of Japanese culture Takeo Doi. In his book *The Anatomy of Dependence (1973)*, which was a bestseller and judged a masterpiece, Doi criticized Benedict’s work, alleging that she underestimated the culture of shame and that the relation between the culture of shame and the culture of sin was underdiscussed. However, he acknowledged that she was right in characterizing Japanese culture as being based on the feeling of shame. He then argued that shame came from being exposed to the public in such a way that one’s *amae* was not satisfied. *Amae* was, according to Doi, the characteristic of a person who is in good favor with, and is able to depend on, those around him or her (Doi, 1973); briefly, this characteristic indicates a protected relationship (Doi, 1974, p. 18). Doi (1973, 1974) also argued that *amae* is desire rooted in the passive affection for the mother, exhibited in early childhood. The Japanese are thus socialized from the first into *amae* as a nucleus of acknowledgment by others and, as they grow, they try to build and maintain relationships with others in such a way as to maintain *amae*. Although *amae* is similar to the English concept of dependence, it has developed in a culturally distinctive way. The structure of Japanese society is based on this and related values. The Japanese language itself reflects this in that it is often easier to express one’s opinions or feelings indirectly and euphemistically (among other examples, the semantic features of such a term as *amae* and the syntactic feature that allows the predicate or the particle of negation to be at the end of the utterance, along with the feature that allows for the ellipsis of various elements of the sentence^4^). Further, among the Japanese themselves, the double standard between *honne* and *tatemae*, or internal and external attitudes, are generally acknowledged and accepted. Unlike Americans, who try to make the two coincide, Japanese often avoid expressing their real intentions in public to support harmony within the group (Doi, 1973, 1974).

Doi’s response to Benedict afforded insight into how the feeling of shame is derived in the psychodynamic process of Japanese everyday life. However, empirical examination was still required to validate the argument. Therefore, it prompted significant discussion among students of Japanese culture and communication. Clancy (1986) did pioneering work in this domain, analyzing the interactions between 2-year-old Japanese children and their mothers to examine their language socialization. She developed a model of how Japanese children are socialized into the distinct Japanese communication style. She noted Doi’s idea of *amae*, and her discussion fundamentally supports its reality as a factor in a Japanese upbringing. In Clancy (1986), interaction between Japanese mothers and children was found to strengthen and reflect cultural beliefs. Mothers often elicit empathy from their children by drawing their attention to the feelings of others to prompt them to perform desired actions. The feelings highlighted in this context can include such emotions as scary, sad, poor, and cute. Mothers, by doing this, draw attention to their own feelings as well as those of a third party, including even unborn children and inanimate objects as having feelings like others (Takada, 2013; Takada and Kawashima, 2016). Clancy (1986) argued that, with such strategies, mothers train their children’s empathy and compassion. As they bring their children into closer consideration of the feelings of others, they are also bringing the pressure of conformity to bear. Thus, empathy and conformity are two sides of the same coin (Clancy, 1986, p. 235). To train her child’s empathy, the mother plants the fear of being laughed at by others. For example, if a child who has behaved inappropriately encounters another person’s disapproval, he or she is expected to feel ashamed. The mother may not specify a grammatical subject or a full sentence on this occasion but may simply say *hazukashii* (shameful or ashamed). With this word, the mother communicates her feeling that the child is *hazukashii* and that the child should feel the same way.

It is not only Japanese culture that is considered to be founded on the feeling of shame. It is also associated with the ethos of other East Asian cultures. According to Lo and Fung (2012), in Taiwan and South Korea, feelings of shame begin in childhood and continue in various forms over the course of life. Additionally, shame is an essential element in morality. Confucianism, which forms part of the common ideological background for Taiwan and Korea in public and educational settings, teaches that human beings can live humbly if they experience shame. In such cultures, children are taught to feel shame from a young age. To shame a young child is to express “a form of love, discipline, and moral teaching that aims to protect the child from future external sanctions” (Lo and Fung, 2012, p. 173). Lo and Fung (2012) analyzed several examples of language socialization regarding shame, such as cases where utterances that included an emotional shame-related term were directed toward children, cases in which gestures customarily associated with shame were used, and cases in which negative assessments that were associated with shame were made. In their analysis, these examples appeared in rebukes, teasing, and expressions of love and intimacy. By employing shame-based communications, a caregiver can guide a child “to reflect upon her own deeds and to develop a sense of right and wrong” (Lo and Fung, 2012, p. 186).

As exemplified in Clancy (1986) and Lo and Fung (2012), studies of language socialization have examined everyday interactions in which children are socialized into a socio-culturally structured universe (Duranti et al., 2012). These studies posit everyday interactions represented in utterance exchanges as the medium of socialization as well as the purpose of socialization across various speech communities. In these works, emotions are regarded as the organizing motive for socialization practices and as the products of such practices. Thus, “work on shaming in the language socialization tradition has documented the verbal routines through which it is enacted, its cultural salience and local meanings, and the ways that young children learn the social and moral norms of a community through shaming” (Lo and Fung, 2012, p. 169).

The present study follows the above research. In particular, it focuses on Japanese caregivers’ use of phrases that include the term *hazukashii*, which can be translated as *shameful*, *ashamed*, *shy*, or *embarrassed*, in accounts of children’s behavior or in teasing children for their behavior. This usage has not been examined to its full extent in previous studies of Japanese socialization. Thus, the term *hazukashii* is considered here with regard to how it emerges within socially situated caregiver–child interactions (CCI) and functions as an organizing force in socialization. This study grounds the existing discussion of the culture of shame and may prompt deeper anthropological study of emotion.

## Materials and Methods

### The Data Set

The data used in this study were collected as part of the longitudinal research project “Cultural Formation of Responsibility in Caregiver–Child Interaction,” which focuses on developmental transitions wherein children’s innate behavioral preferences are shaped into coordinated patterns of interaction to meet the expectations of both caregiver and child (Takada et al., 2016). The author directed this project from 2007 to 2012 and supported follow-up projects^5^. The data were collected in the Kansai region of Japan.

Commencing in 2007, the research team began to visit 17 middle-class families with children aged 0–5 years with the aim of collecting data. The families were chosen from among those who expressed interest in the Kyoto University Child Development Research Group^6^. All families used the Kansai dialect for daily communication. A researcher and a videographer visited each family at home for approximately 2 h per month to record the interactions between the child(ren) and caregiver(s) in their natural settings in that family. Most families consisted of caregivers and more than one child, as one of the project’s objectives was to elucidate how older siblings developed a sense of responsibility. Some mothers who participated in this study were pregnant at the time the data were collected. This is relevant to the analysis because an unborn child may be the subject of conversation and a participant in an interaction. In total, approximately 410 h of video were recorded, and all basic verbal and non-verbal behaviors were transcribed to yield the data set. Although there was no intention of creating a balanced sampling, the data set nevertheless reflect the everyday life of ordinary Japanese families with young children.

### The Collection of *Hazukashii*

Using the search system^7^ created for the project, I extracted parts of transcripts that contain the term *hazukashii*. Then, I examined the extracted sections and made a collection of 337 phrases that included *hazukashii* (see Table 1 for details). Then, I checked the flow of interaction within the transcripts. Following this, I chose several interesting scenes and made more detailed transcripts of them, using the film recordings. Some scenes featured more than one phrase including *hazukashii*.

**Table 1:** Occurrence of phrases including *hazukashii* by family.

Family	Observed time length (hour:min)	Phrase (*n*)	Phrase (*n*)/hour	Phrase (%)
TM	73:09	87	1.2	26%
SA	70:09	30	0.4	9%
KT	35:18	99	2.8	29%
SB	34:21	10	0.3	3%
KB	24:21	7	0.3	2%
FM	23:37	14	0.6	4%
SG	23:16	40	1.7	12%
SK	22:16	3	0.1	1%
ST	21:43	3	0.1	1%
MB	16:31	6	0.4	2%
KJ	13:31	1	0.1	0%
UZ	13:20	3	0.2	1%
OM	12:28	20	1.6	6%
SI	12:11	3	0.2	1%
HK	7:52	6	0.8	2%
TK	4:05	1	0.2	0%
SY	1:43	4	2.3	1%
Total	410:00	337	0.8	100%

This paper reports on the preliminary analysis of that collection and uses examples from three families, referred to by the initials TM, KT, and SA in the excerpt titles. In the excerpts transcribed below, each line includes the original Japanese utterance^8^, word glosses^9^, and an English translation. Proper names are given as pseudonyms in the form of initials or are modified for the sake of anonymity.

### Interaction Analysis

Interaction analysis was used to examine gestures and speech in these excerpts, using analytical concepts derived from conversation analysis (Schegloff, 2007; Sidnell and Stivers, 2013) and language socialization studies (Schieffelin and Ochs, 1986; Duranti et al., 2012). Interaction analysis is an empirical method of determining why a given action is performed at a specific place and time, done by deconstructing the sequential organization of the interaction (i.e., by clarifying the mutual relevance of adjacent actions: Schegloff, 2007; Nishizaka, 2008). This approach explains not only how certain actions are taken within a particular socio-cultural context but also how those actions alter the context. This method is thus a variant of the integrative approach to the study of human sociality, which combines the analysis of situated social interaction with ethnographic procedures (Demuth and Fatigante, 2012).

## Results

In Japanese CCI, both caregivers and children use phrases including the term *hazukashii*. Such phrases can be used to describe the child’s action or state, those of the caregiver, or of other figures that appear in the interaction in whatever form. In our data set, caregivers frequently uttered phrases to young children that included *hazukashii*, even at a very early age. Table 1 shows the distribution of the use of such phrases by family. It indicates that phrases including *hazukashii* were broadly observed in all families, although the actual rate of occurrence varied among families (averaging 0.8 times per hour).

Phrases including *hazukashii* were used more frequently by caregivers than by children. They were often used in accounting children’s hesitation to perform an appropriate action (i.e., being shy or embarrassed).

### *Hazukashii* as Accounting

**FIGURE 1:**
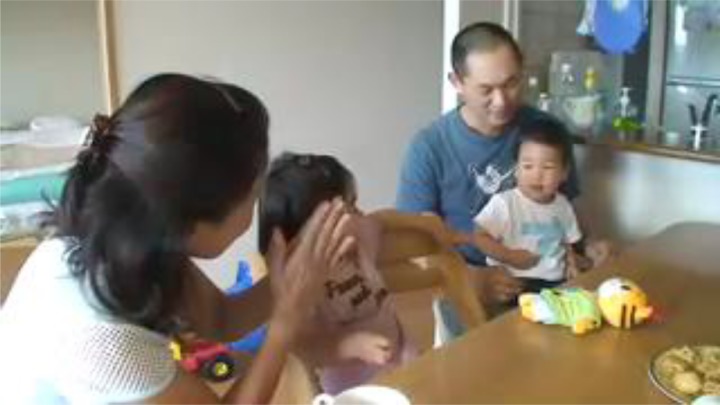
M prompts T to clap his hands (written informed consent was obtained from the depicted adults and parents of depicted children for the publication of these images).

The excerpt below is drawn from interactions involving the male infant T, who was 12 months old, and his parents and older sister, N. During the excerpt, T is being held on his father’s (F) lap. Both are facing toward a large, set dining table. N sits on the chair to the right of F and T, and the mother (M) is sitting in a chair to the right of N (Figure 1). On the other side of the dining table, the researcher (R) is sitting (she does not appear in the video). Excerpt 1 begins when the mother addresses T by his name, and T replies with a vocalization and a smile.^10^ In the transcription of the excerpt, an arrow (→) indicates an utterance that contains *hazukashii*.

**Excerpt 1 Pachi pachi (TM_K080628_1)**

**T (1:0), N (3:1), M (mother), F (father), R (researcher)**

1 M: [pachi pachi pachi:: wa?
  SSW SSW SSW TOP
  **[((can you make a hand gesture of)) pachi pachi**
  **pachi::?**
2 F: [hu::n
  IJ
  **[hu::m**
  **((T stops smiling and turns his eyes away from M.))**
3 M: pachi pachi::
  SSW SSW
  **pachi pachi::**
4 R: [hu::n
  IJ
  **[hu::m**
5 M: [pachi pachi:: shite.
  SSW SSW do-TE
  **[((make a hand gesture of)) pachi pachi::.**
6 M: ° are:¿°deki hen¿
  IJ can NEG
  **°oh:¿°((you)) can’t ((do it))¿**
7 M: pachi pachi[:::
  SSW SSW
  **pachi pachi [:::**
  **((T redirects his gaze toward M.))**
8 N: [deki hin?
  can NEG
  **[((you)) can’t ((do it))?**
9 M: pachi pachi: shite
  SSW SSW do-TE
  **((make a hand gesture of)) pachi pachi::**
→10 F: hazukashii no¿
  shy Q
  **((are you)) *hazukashii*¿**
  **((T turns his eyes away from M and N and looks ahead.))**
11 R: hh[h
  **hh[h**
12 F: [hh
  **[hh**
13 M: nantonaku itteru koto wa tsutawatteru kanji ga(0.2) somehow saying thing TOP conveying feeling NOM** it seems that somehow he gets what I say(0.2)**

In line 1, M prompts T to clap his hands by speaking a phrase that combines the onomatopoeia *pachi pachi pachi* and the particle *wa* (delivered in a rising tone, indicating a question form), which designates topicalization (Figure 1). Prompting is a subcategory of directives (Takada, 2013; Takada and Endo, 2015), which are defined utterances “intended to get the listener to do something” (Goodwin, 2006, p. 107). A similar onomatopoeia is used in lines 3, 5, and 7, and all of these utterances are combined with clapping. In addition, along with M’s utterance in line 1, F gives an utterance that sounds like an imitation of the preceding vocalization of T (line 2). Seeing M’s prompting action, T stops smiling and turns his eyes away from M.

Almost simultaneously, M prompts T to clap (lines 3 and 5). At this point, R makes an interjection that is similar to F’s interjection in line 2 (line 4). However, T does not react to these actions. M then makes a request in the form of negation (line 6) and then makes prompts again (line 7). T then redirects his gaze toward M as if reacting to M’s onomatopoeia and clapping. N makes a request in the form of a negation “*deki hin?*” [“((you)) can’t ((do it))?”], which is similar to the previous utterance by M, and claps (line 8). This request falls into the subcategory of directive (Takada, 2013; Takada and Endo, 2015). T looks at M and N again, smiles faintly, and begins to clap his hands in a half-hearted manner, but he quickly stops. Then, M prompts in the form of a request (line 9).

In the above interactions, M and N repeatedly issue modified directives, which creates a rhythm in their interaction, as they monitor T’s behavior. They thereby try to make T clap his hands happily. However, T does not react appropriately to these directives.

**FIGURE 2:**
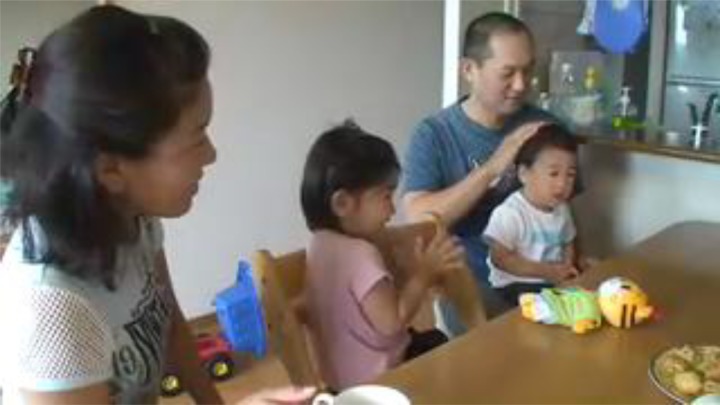
F gently strokes T’s head and giggles (written informed consent was obtained from the depicted adults and parents of depicted children for the publication of these images).

Then F, who is holding T on his lap, asks him “((are you)) *hazukashii*¿” (line 10). Simultaneously with this utterance, T turns his eyes away from M and N and looks ahead, and looks ahead to where the researcher is sitting. F then gently strokes T’s head and giggles (Figure 2). The utterance in line 10 provides an account, which attributes the lack of sufficient response by T to the preceding directives to T’s emotional state of *hazukashii*. This also works as an assertion that the lack of response does not imply inability (e.g., that he is too young to understand the utterances) or any intention to resist the directives (e.g., that he does not want to clap his hands). Here, *hazukashii* means something like being embarrassed or shy, though it should be noted that the English word shy can be an attitude or a trait, while the term *hazukashii* here indicates a transitory emotional state derived from particular circumstances. Thus, the equivalent expression would be to be embarrassed. This account appears to be accepted by R and M. Immediately R laughs, showing agreement with F (line 11). F laughs together with R (line 12). Finally, M comments that T has understood the preceding directives (line 13).

The following excerpt involves the same family as in Excerpt 1, and the phrase including *hazukashii* is used to account for the behavior of the child. About 2 months have passed since the recording of Excerpt 1. The mother (M) is standing inside the kitchen, with N standing on a chair across the bar counter. In front of N, there is a large dining table, as in Excerpt 1. The father (F) sits opposite the mother, as seen by N (however, he is not on screen). Before this excerpt begins, N and his parents are speaking of whether cicadas are frightening or cute. The excerpt begins as M gives an iced coffee to C, the camera operator filming the video.

**Excerpt 2 Please give it (TM_K080907_2)**

**N (3:3), M (mother), F (father), C (camera operator)**

1 M: =a, so- kore C san ni douzo shite(nkai) (1.2)
  IJ it this Mr. C DAT please do+TE
  **=oh, please give i- this to Mr. C**
2 M: oniisan ni douzo (tte)
  Brother DAT please+TE
  **(saying) “*douzo*” to him**
  **((N taps the table and then brings the mug to her mouth by her right hand.))**
3 M: sore Naho no.
  It name LK
  **it’s Naho’s.**
4 M: nhu
  IJ
  **nhu**
  **(4.0)**
5 M: motte ikeru¿ Naho
  grab+TE can.go name
  **can you bring it to him¿ Naho**
→6 F: iya, hazukashii ka [na¿
  no shy Q PP
  **um, ((you are)) *hazukashii*, aren’t [you¿**
→7 M: [hazukashii n ka
  shy LK Q
  **[((are you)) *hazukashii***
8 M: [motte ike nai¿ kore °motte ikeru?°
  grab+TE can.go NEG this grab+TE can.go
  **[can’t you bring it to him¿ can °you bring this to him?°**
9 F: [he(h)he(h)he(h)he(h)
  **[he(h)he(h)he(h)he(h)**
→10 F: (sore)hazukashii[yarou
  it shy TAG
  **((you)) would be hazukash[ii**
→11 M: [hazukashii na
  shy PP
  **[((you are)) *hazukashii***
  **(9.0)**
12 F: erai genki ga=
  greatly cheer NOM
  **((she)) fairly ((lost her)) cheer=**
13 M: =ee¿
  IJ
  **=what?**
14 F: =ima made no genki ga dokka i tta
  now until LK cheer NOM somewhere go PST
  **=her cheer ((that she had)) just now went somewhere**
15 M: ee¿
  IJ
  **what?**
16 F: genki ga dokka i tta=
  cheer NOM somewhere go PST
  **her cheer went somewhere=**
17 M: =honma yane:(h)
  right PP
  **=that’s true(h)**

**FIGURE 3:**
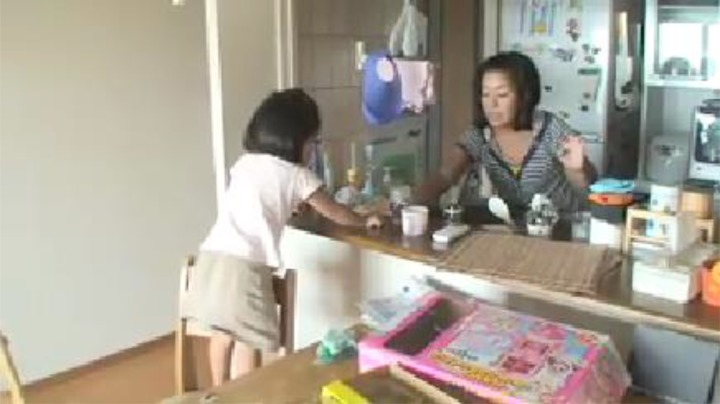
M asks N to pass the glass to the camera operator (written informed consent was obtained from the depicted adults and parents of depicted children for the publication of these images).

N leans her body on the bar while looking at the iced coffee glass. M asks N to pass the glass to the camera operator (line 1; Figure 3). The phrase *douzo* (please) is frequently used when Japanese caregivers prompt children to do something. Here, M prompts N to perform a chore, namely, bringing a glass of iced coffee to the guest. Following this, N pulls her body slightly upright. Watching this, M makes prompts again, using the utterance *oniisan ni douzo (tte)* [(saying) “*douzo*” to him] (line 2). In this second prompting, the proper name Mr. C is replaced by *oniisan*, a title derived from the kin term for elder brother. The latter is a friendlier expression to use in referring to N. Furthermore, the quotation marker *tte* emphasizes that the utterance is a prompting.

Then, N looks at M and bangs on the table (in the video, the sound is clearly heard) to indicate her resistance to the directive. She then brings a mug of iced tea, which is set next to the glass, to her mouth. Immediately M acknowledges this action, saying, “it’s Naho’s” (line 3). Looking back, N continues to drink tea. The mother interjects “nhu” (line 4) to draw N’s attention. Then, N looks at M again while putting the mug to her mouth and puts the mug back onto the counter.

Watching this, M reiterates her directive (line 5). This utterance takes the form of a request, which is a sub-category of a directive and a stronger expression than prompting (Takada, 2013; Takada and Endo, 2015). The expression *motte ikeru¿* (can you bring it to him¿) conveys both whether N has the ability to carry out the action and whether she has the intention of performing the action. Furthermore, by using the name Naho, she makes it clearer that N is the addressee of the directive. Both communications tend to increase the pressure of the directive.

However, N shows no sign that she intends to pick up the glass. The lack of N’s appropriate response (i.e., second pair part) to the mother’s directives (i.e., first pair part) indicates that the adjacency pair is incomplete and, thus, renders the child’s non-compliance visible. Seeing this, F gives an account for N’s series of actions, saying, “*iya, hazukashii ka na¿*” [um, ((you are)) *hazukashii*, aren’t you¿], which can be understood to mean, “you are embarrassed, aren’t you?” (line 6). The interjection *iya* (um) at the beginning of this utterance indicates that he does not take the lack of N’s appropriate response to the mother’s directives as non-compliance. Moreover, it projects that another account for N’s series of actions will follow. Then, F asserts that N has not given the iced coffee to C because she is *hazukashii*. This term is used here in the same meaning as in Excerpt 1, namely, being shy or embarrassed. That is, F is attributing the reason for N’s failure to act properly to a temporary emotion caused by the situation at the moment. The interjection is provided as a more understandable interpretation that interprets N’s behavior as a lack of reaction rather than non-compliance. Furthermore, *kana* appears in this utterance, a final particle that indicates a question or confirmation. Thus, judgment on the pros and cons of the account is directly entrusted to N and indirectly to other hearers. Partially overlapping with this utterance, M repeats F’s comment (line 7). This is done with a smile and in a whispering voice as M reaches for the glass containing iced coffee. This acknowledges the account of the preceding father’s utterance.

Then, M repeats the request twice more (line 8) and places the glass slightly closer to N. The first of these iterations of the request includes the negative question form *motte ike nai¿* (can’t you bring it to him¿), which is intended to elicit N’s voluntary action. In addition, F laughs at the same time as this is said (line 9). While looking at M, however, N picks up the mug containing tea without showing any sign of reaching for the glass of iced coffee. F acknowledges this, saying “((you)) would be *hazukashii*” (line 10). Note that *yarou*, which marks a tag question, is used here. This word strengthens the father’s epistemic stance (Heritage, 2012), confirming the correctness of the account with a greater degree of certainty than the utterance in line 6. Overlapping with this, M repeats F’s utterance one more time (line 11). Here, the final particle *na*, which indicates confirmation, follows immediately after the term *hazukashii.* This is designed to confirm that the account is correct. N does not reply, however, but continues to drink tea while watching M.

Then, F says that N’s energy has gone somewhere (lines 12, 14, 16). M asks for a repair (Kitzinger, 2013) at lines 13 and 15, which may indicate that the prior utterances (lines 12, 14) are difficult to hear. Finally, she exhibits agreement, saying, “that’s true,” with laughter (line 17).

### *Hazukashii* as Teasing

In our dataset, the term *hazukashii* also frequently occurred in the context of teasing a child or saying that certain action(s) carried out by the child are inappropriate in relation to social norms (i.e., shameful or something to be embarrassed/ashamed about).

In the next example, the girl A, 2 years and 9 months old, is watching a video, taken while A was still an infant, with her mother (M) in her last month of pregnancy (on the TV screen, children, including baby A, and a woman, who appears to be the nursery teacher, are seen). M cautions A to take a step back. A turns to M, rising up on her knees, and tries to move back as instructed. The excerpt begins from there.

**Excerpt 3 It’s a baby (KT_A080310_2)**

**A (2:9), M (mother)**

1 A: a:::
  IJ
  **a:::**
2 M: h akachan ya
  baby PP
  **h it’s a baby**
3 A: a:[: a a a
  IJ IJ IJ IJ
  **a:[: a a a**
4 M: [akachan ya.
  baby PP
  **[it’s a baby.**
5 A: a::[:
  IJ
  **a::[:**
6 M: [akachan ya.
  baby PP
  **[it’s a baby.**
7 A: a::[:
  IJ
  **a::[:**
8 M: [akachan donnan nan no?
  baby how become Q
  **[what’s the baby going to do?**
9 M: akachan donnan nan no?
  baby how become Q
  **what’s the baby going to do?**
10 A: aa, iya ya.
  IJ no PP
  **oh, no.**
11 M: are akachan ja nai. hh ima(h) demo(h)
  IJ baby PP NEG now but
  **oh, it’s not a baby. hh but just now(h)**
  akachan(h) natta(h) yaro.
  baby became FP
  **you behaved(h) like(h) a baby**
→12 M: iya(h) aka(h)chan(h) natta(h), ha(h) zu(h)ka(h)shii(h).
  wow baby became shameful
  **wow(h), ((you)) behaved(h) like(h) a(h) baby(h),**
  **((you should feel)) ha(h)zu(h)ka(h)shii(h).**
13 M: akachan [(nattan)
  baby become-PST-Q
  **((you)) (behaved) as a ba[by**
14 A: [aa::
  IJ
  **[aa::**
15 A: a aha aha aha utta.
  IJ IJ IJ IJ hit-PST
  **a aha aha aha ((I)) hit it.**
16 M: hora hora akachan ni.
  IJ IJ baby DAT
  **hey, hey, ((you behaved like)) a baby.**
17 M: akachan natta?
  baby become-PST
  **((did you)) behave as a baby?**

**FIGURE 4:**
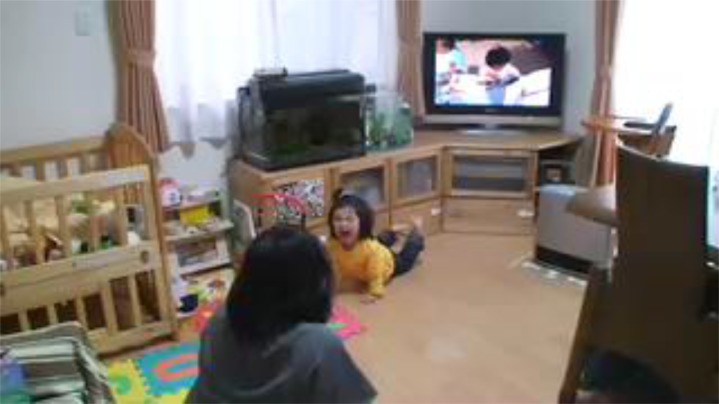
A begins to pretend to cry like a baby (written informed consent was obtained from the depicted adults and parents of depicted children for the publication of these images).

**FIGURE 5:**
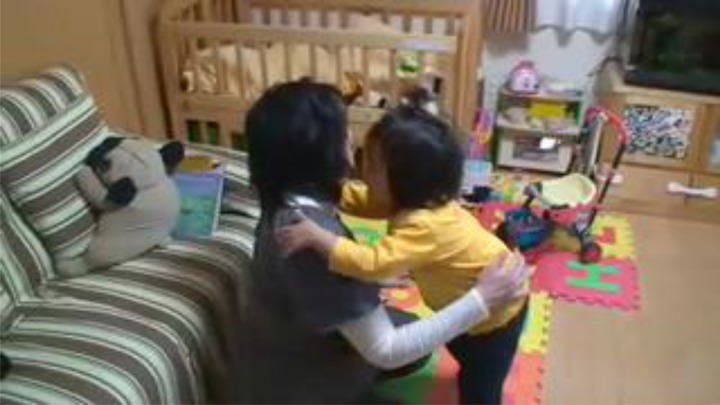
A and M hug each other (written informed consent was obtained from the depicted adults and parents of depicted children for the publication of these images).

A loses her balance and her hands strike the floor. A immediately begins to pretend to cry like a baby (line 1; Figure 4). M immediately breathes in and remarks “it’s a baby” (line 2), acknowledging the change in the footing (Goffman, 1981) of A’s utterances. The mother says that A was inspired by baby A on the screen to pretend to be a baby. Perhaps this (A pretending to be a baby) is also related to the fact that M is in the last month of her pregnancy and A is conscious of the baby who will soon be born. Then, A performs exaggerated mock crying (line 3). Overlapping with this, the mother repeats, “it’s a baby” (line 4). As similar utterance exchanges are reiterated in lines 5 and 6, A goes to M, and they hug each other (Figure 5). M holds A gently. In these interactions, there is a playful atmosphere between them.

**FIGURE 6:**
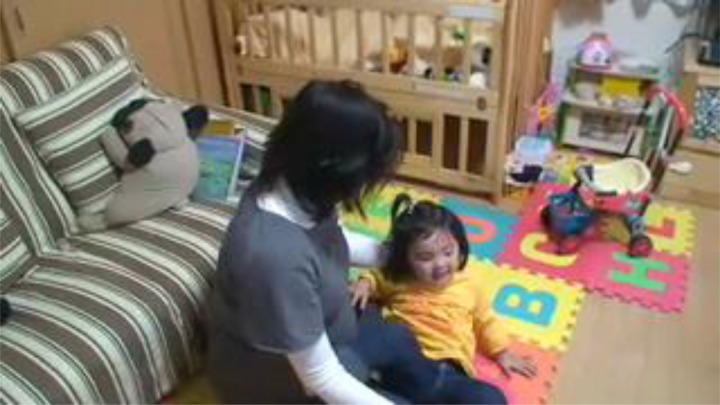
A releases herself from M’s embrace (written informed consent was obtained from the depicted adults and parents of depicted children for the publication of these images).

Addressing A, who continues to pretend to cry, M formulates the open question, “what’s the baby going to do?” twice (lines 7–9). At the same time, M holds A under her arm as if she will feed her like a baby. These open questions act as a prompt for A to upgrade the pretense play of being a baby by adopting a posture in which A is held (i.e., performing the role of being breastfed). The situation is embarrassing for A because, first, open questions, which require selecting the words included in an answer from various candidates, are generally difficult for young children, who are not yet adept at using language (Takada and Kawashima, 2016). Additionally, M’s utterances here are provocative as they imply that A is being tested as to whether she can appropriately upgrade the pretense play of being a baby. A then changes the footing of her utterance again, saying, “oh, no,” and releases herself from M’s embrace (line 10; Figure 6). This utterance and behavior indicate that A rejects the preceding open question.

M notices this and checks her understanding that A has behaved like a baby (line 11). M then offers the following comment, while laughing: “((you should feel)) *ha(h)zu(h)ka(h)shii(h)*” (shameful or ashamed).^11^ This utterance is designed as teasing A by laughingly pointing out the gap between the role played by A and A’s usual behaviors.

While M is saying this, A gives M a hug. The mother teases A, saying, “wow(h), ((you)) behaved(h) like(h) a(h) baby(h),” while laughing, and then she makes the following assessment (Goodwin and Goodwin, 1987) of A’s action in line 12: “((You are)) *ha(h)zu(h)ka(h)shii(h)*.” Simultaneously, M pats A on the back repeatedly. *Hazukashii* is here used in a meaning that is closer to “shameful” or “ashamed,” although it is used in a playful context. In other words, the mother is teasing A, saying that A, who has acted younger than her actual age, should feel ashamed, and M also feels this to be shameful. This utterance exchanges dissolve the friction in the interaction. The mother repeats the question, “((you behaved)) as a ba[by,” confirming her feeling in line 13. A then begins her mock crying again (line 14). However, this mock crying gradually shifts to an ordinary, embarrassed vocalization, and then A explains why she was crying (because she hit her leg on the ground) (line 15). In response, the mother confirms that A has acted like a baby (lines 16 and 17), but these utterances are made in a gentle and ordinary tone of voice; it appears that the teasing atmosphere of the preceding utterance is now gone.

Let us examine another example of teasing. Below, the woman M, her son Y, 4 years and 1 month old, and her daughter B, 8 months old, are having lunch. M is sitting in front of Y at a table. B sits on M’s left oblique front. Before the excerpt begins, Y is eating by himself. Then he gradually loses his appetite. Seeing this, M begins to serve him his food directly, using her chopsticks, as she also has been doing for B. The excerpt begins there.

**Excerpt 4 Girls will laugh at you (SA_Y090612_2)**

**B (0:9), Y (4:1), M (mother)**

1 M: soko wo fuki, kore de
  there ACC wipe this by
  **wipe there by this**
  **((M hands out a tissue to T.))**
2 Y: au::
  IJ
  **au::**
→3 M: kitanai de. hazukashii yo, sore.
  clumsy PTC shameful PP it
  **it’s untidy. it’s *hazukashii***
  **((M wipes Y’s face by a tissue.))**
4 M: hora hora hora tobideten de, akan na::hh
  IJ IJ IJ runing over PP not good PP
  **hey hey hey ((something)) is running over, it’s not goo:d hh**
  **((M wipes Y’s nose by a tissue.))**
  **((5 lines are omitted))**

10 M: hai.
  IJ
  **here you are.**
  **((M feeds a piece of meal to Y.))**
11 Y: de.
  IJ
  **and**
→12 M: hazukashii, youchien demo kouyatte ( ) tabe
  shameful kindergarten also like this eat
  **it’s *hazukashii*. what will you do**
  sashi te moratteru toko sirare tara dou suru:?
  CAU TE give TE be place be found COND how do
  **kindergarten friends see you being fed like this?**
  **(2.4)**
13 M: [naa.
  IJ
  **[hey.**
14 Y: [(houhun na).
  **[( ).**
  **(2.0)**
15 M: na.
  IJ
  **hey**
16 M: Kiko chan toha: Yuki chan toka: Hana chan toka,
  name DIM and name DIM and name DIM and
  **little Kiko, little Yuki, little Hana**,
  Kano chan ni: Yasu kun tte mada tabesasete
  name DIM DAT name DIM TE still eat CAU TE
  **and little Kano, may say “Yasu is still fed**
  moratten no: tte
  give PP QT
  **((by his mother))”**
17 M: dou suru:?
  how do
  **what do you do:?**
18 Y: **((Y greatly swings the head for four times with a**
  **smiley face.))**
→19 M: minna hitori de tabete han noni, hazukashii
  all alone by eat TE HON though shameful
  **all of your friends are eating by themselves though. ((you’re)) *hazukashii:***
  **(2.4)**
20 M: onnanoko ni waraware chau wa yo.
  girl DAT be laughed PER PP PP
  **girls will laugh at you.**
21 Y: **((Y weaves his hands on his head.))**
22 Y: (u:tsusu) (ujanshan)
  ( ) ( )
  **((Y looks at B, and then points to B by the pointing finger of his right hand.))**
23 Y: shabo:n natteru
  SSW become
  **it’s like shabo:n**
  **((Y raises the pointing finger, and then shakes it.))**
24 M: shabon?
  SSW
  **shabon?**
  **((M looks at Y’s face.))**
15 Y: kondo.
  next time
  **next time.**
26 M: (kero).
  **( ).**
  **((M picks up the chopsticks.))**
27 M: shobon ka¿
  SSW Q
  **you mean shobon¿**
28 Y: un.
  IJ
  **yeah.**
  **(1.4)**
29 M: gochi sho: sama deshi ta.
  nice meal HON POL PST
  **thank you for the meal.**
  **((M puts her palms together while having meal in her mouth.))**
30 Y: **((Y puts his palms together))**

Seeing the crumbs around Y’s mouth, M stretches out her right hand, holding a tissue, saying “wipe there by this” (line 1). Y frowns and moves his body backward, saying “au::” (line 2). She then says “*kitanai de. hazukashii yo, sore*” (“it’s untidy. it’s *hazukashii*”) (line 3). *Hazukashii*, which here means shameful, is used to call Y’s attention to his poor eating manners and motivates him to eat in a more decorous way. M uses a normal tone of voice, and there is little nuance of condemnation. Y moves his body as if to dodge the offer of the tissue, but then he brings his face close to M’s hand, and M begins to wipe his face. While wiping, she says, “hey hey hey ((something)) is running over, it’s not goo:d hh,” and smiles (line 4).

**FIGURE 7:**
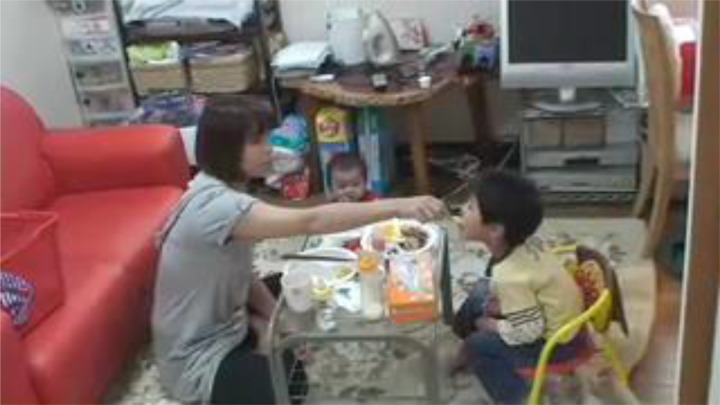
Y brings his face close to M’s hand and eats the piece of food (written informed consent was obtained from the depicted adults and parents of depicted children for the publication of these images).

Then, Y lists something he remembers, while repeatedly placing the index finger of his left hand on the palm of his right hand (these utterances are omitted in the transcript). Then, M feeds Y a piece of food, saying, “here you are” (line 11), so as to motivate Y to eat in a tidy manner. Y immediately brings his face close to M’s hand and bites the piece of food (line 12; Figure 7). Subsequently, the interaction shifts into a more playful mood. While watching Y being fed, M says “*hazukashii, youchien demo kouyatte () tabe sashi te moratteru toko sirare tara dou suru:?*” (it’s *hazukashii*. what will you do if your kindergarten friends see you being fed like this?) (line 12). Here, M assesses Y’s behavior (i.e., that M is serving him with her chopsticks, although Y is already 4 years old) as *hazukashii*. Here, *hazukashii* can be translated as shameful (because the behaviors are overly childish). M highlights this meaning by calling on him to imagine how his kindergarten friends would find those behaviors. However, M is smiling during the latter part of this utterance. Thus, it is evident that M is teasing Y for his behavior. After making inquiries twice (lines 13 and 15), M pronounces the names of four of Y’s friends. She then changes the footing of her utterance, giving their words in reported speech, “*Yasu kun tte mada tabesasete moratten no: tte*” (say, “Yasu is still fed ((by his mother))” (line 16). She then reiterates her inquiry (line 17). That is, M continues to tease.

**FIGURE 8:**
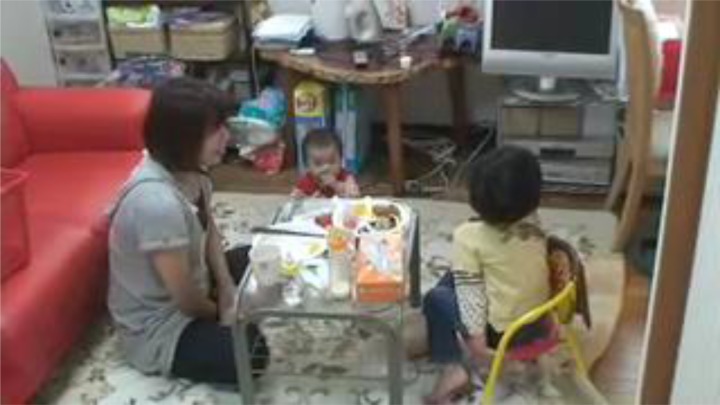
Y shakes his head, while slightly smiling (written informed consent was obtained from the depicted adults and parents of depicted children for the publication of these images).

Here, Y avoids making a clear response, waving his hands and feet with food in his mouth. As in Excerpt 3, it appears that open questions are difficult to answer for young children who are not adept with language. In line 18, Y shakes his head four times to the right and left, while slightly smiling (Figure 8). These head movements may indicate a denial or rejection of M’s preceding utterances. However, Y does this in a rhythmic and exaggerated manner. Thus, it resembles a choreographed dance rather than a simple denial or refusal. Additionally, Y makes these motions with a smile. Overall, he demonstrates that he understands M’s utterances as teasing.

It seems that Y’s reaction is insufficient for M. In line 19, she upgrades the teasing. That is, M emphasizes the difference between Y and his friends, saying, “*minna hitori de tabete han noni*,” (all of your friends are eating by themselves though). Furthermore, she assesses Y’s behavior again as *hazukashii*. After that, however, following a silence of 2.4 s, during which Y does not sufficiently respond to this utterance, M again further upgrades the teasing, saying, “*onnanoko ni waraware chau wa yo*,” (girls will laugh at you) (line 20). This utterance combines a gender categorization with the previous norm of psychological/behavioral maturity and, thereby, strengthens the impact of *hazukashii*. That is to say, in addition to its being considered *hazukashii* for 4-year-old Y to be fed by his mother, it is doubly *hazukashii* for the boy Y if that behavior were to be known by the girls in his kindergarten class. In other words, the fact of Y’s “being fed” is assessed in terms of the following norms: that a 4-year-old child should be able to eat a meal properly by himself/herself and that boys should not be laughed at by girls. Additionally, a more direct negative assessment is made with reference to the specific action of girls’ laughter or ridicule. Both of these emphasize the inappropriateness of Y’s previous behavior through teasing.

**FIGURE 9:**
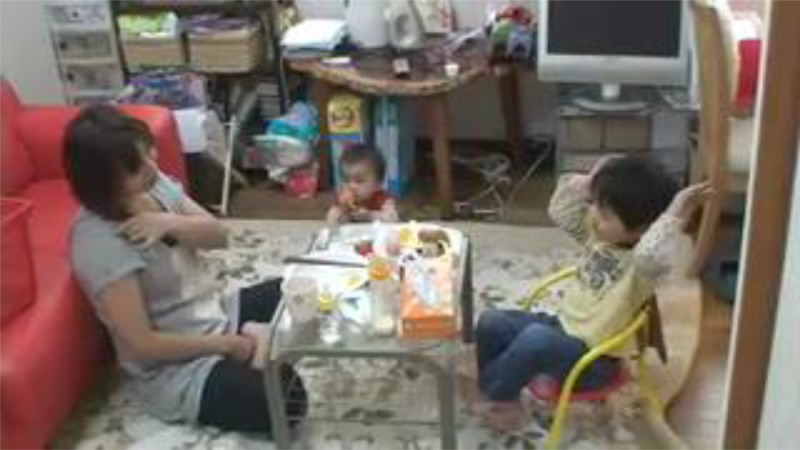
Y waves his hands above his head (written informed consent was obtained from the depicted adults and parents of depicted children for the publication of these images).

Listening to this, Y waves his hands above his head and makes non-verbal interjections (line 22; Figure 9). This display denotes resistance to M’s utterance. He then points to B with the index finger of his right hand (line 23) and says, “it’s like *shabo:n*,” while raising his index finger and shaking it (line 24). He thereby avoids reacting to M directly, that is to say, mitigating the face-threatening situation (Goffman, 1981; Brown and Levinson, 1987) and, instead, expressing his reaction to his younger sister, who is still an infant. Then, M initiates a repair for the unintelligible part of Y’s prior utterance (i.e., *shabo:n*) (line 24). After three lines, M proposes a candidate answer for the repair, saying, “*shobon ka¿* (you mean *shobon*¿)” (line 27). *Shobon* is a customary expression that indicates a state of discouragement. Y acknowledges this (line 28). Then, M closes the dining activity with a customary utterance and gesture (line 29). Y replies with the same customary gesture (line 30).

## Discussion

The term *hazukashii* is used in Excerpt 1 during the latter part of the activity, where T is being prompted to perform the socially desirable act of clapping his hands in front of the researcher, present at the time of filming. The term *hazukashii* in Excerpt 2 is introduced in the latter part of a sequence of repeated directives to pass a drink to the camera operator. These interactions both involve a person who is not usually included among the family members and does not know them well, and a child is being asked to act in a certain way toward that person. Thus, the outsider present at the time of filming is treated as the addressee or hearer (Goffman, 1981) of the word.

In Excerpt 1, a caregiver uses a phrase containing the term *hazukashii* to describe the fact that the child does not perform appropriate actions in relation to given social norms. In this way, he presents a candidate account that is due to embarrassment caused by the immediate situation. In Excerpt 2, the phrase in which the father employs the term *hazukashii* describes an omission to act by a child, which is deemed inappropriate. Here again, the father presents a candidate account that the failure is due to embarrassment (line 6). Subsequently, the mother acknowledges this assessment (line 7). Then, in response to the fact that the behavior of the child does not improve, the father presents the account once more, again including *hazukashii* (line 10), and the mother partially repeats it, thereby confirming the account (line 11). In all of these cases, the term *hazukashii* is used with a meaning close to that of being shy or embarrassed.

Whether the child hesitates to conduct an appropriate action or performs an act that can be deemed inappropriate, the term *hazukashii* in these excerpts indicates the account for the action or omission in relation to the given context and functions to make the action or omission understandable. In this way, an actor whose action is deemed *hazukashii* is given the opportunity to justify, modify, or repeat preceding actions in a more appropriate manner as a next action. This provides the child with early opportunities that enhance *amae*, that is, an actor presumes upon the recipient’s willingness to cooperate, empathize, and intuit what he/she has in mind (Doi, 1974). That is to say, the cooperative and empathetic attitude of the caregiver becomes visible to the child through the demonstration of understanding in relation to his/her inappropriate or inadequate actions. Therefore, phrases including the term *hazukashii* serve as a useful tool for the language socialization of children in the talk-in-interaction process among Japanese speakers.

In Excerpt 3, a phrase containing *hazukashii* was employed to note the gap between what A does, inspired by the given context in the video, and A’s ordinary behavior (line 12). A is in a somewhat distant context from the ordinary routine of family communication. It is difficult for her to behave properly in this context. In this case, the mother teased A by pointing to her being overly childish. In a teasing context, *hazukashii* is used with a meaning that is close to shameful, which is an assessment.

In Excerpt 4, the mother uses the term *hazukashii* in the sense of shameful to draw Y’s attention to his clumsy way of eating (line 3). However, the mother’s interest shifts shortly thereafter to Y’s overly childish behavior (allowing his mother to serve him food with her chopsticks). She mocks Y’s behavior, using the term *hazukashii* in the meaning of shameful or feeling ashamed (lines 12, 19). By doing this, the mother is repositioning Y’s inappropriate behavior in the context of play. Here, she highlights the term *hazukashii* by introducing outsiders (here, kindergarten friends) to the participation framework of interaction. The attribution of others’ emotions or feelings in utterances is one way that Japanese caregivers teach children to be sensitive to others (Clancy, 1986, p. 233). Moreover, by introducing a virtual third party, Japanese caregivers often create a playful and theatrical participation framework, which is usually less face threatening and can elicit socially desirable behaviors on the part of the child (Takada, 2013; Takada and Kawashima, 2016). Such utterance exchanges would provide the child with lived experiences to enable developing intersubjectivity and alterity (Demuth, 2013).

Children who are teased for the inappropriateness of their behaviors may develop their play further, or they may correct their previous action to affiliate with socially shared normativity. In Excerpt 4, however, Y does not immediately give the proper response, showing an ambiguous attitude. For this reason, the mother gradually upgraded the teasing (lines 16, 19, 20). Y’s responses also changed accordingly and, eventually, he joined the theatrical play set up by his mother, bringing in his infant sister B.

According to Clancy (1986, pp. 237–238), teasing is used to train children’s conformity by planting the fear of being laughed at by others. Benedict (1946) also discussed the importance of early teasing in that it nurtures the fear of ridicule in the child’s later life. However, in this study, teasing is primarily in a playful context, and the orientation to conformity training is not strong. Rather, it is motivated to promote interactions *in situ* in a cooperative and pleasant atmosphere. Lo and Fung (2012) also found that a considerable proportion of spontaneously occurring shaming events at home are in a playful key (Lo and Fung, 2012, p. 181). Relative to other negative assessments that directly mark the speaker’s intentionality, teasing in a playful context is less threatening to the child’s face (Brown and Levinson, 1987). Teasing also facilitates a multiplicity of frames in conversations between children and caregivers.

In all of our cases, the caregivers attend to various semiotic fields (e.g., direction of gaze, facial expression, posture, intensity of action, seriousness of performance, and footing of action) (Goodwin, 2000), while monitoring minute changes in the child’s action and situation associated with the child. Caregivers thereby connect diverse semiotic resources to display an empathetic attitude to the child (Excerpts 1 and 2) or create a cooperative and amusing atmosphere (Excerpts 3 and 4). In these ways, a phrase containing the term *hazukashii* indicates that the child did not perform an appropriate action with respect to the context of the specific social situation. Consequently, the different meanings of the term *hazukashii* (i.e., embarrassed, shy, ashamed, or shameful) are made available to the child in each context. In the course of the child’s development, the meaning of ashamed, or shameful tends to appear relatively late, on the basis of the child’s understanding of the meaning of embarrassed, or shy. In other words, developmentally, the people surrounding the child initially indicate/suggest what *hazukashii* means, and they then gradually start expecting that the child also feels *hazukashii*. Therefore, phrases including the term *hazukashii*, or emotional terms in general, function as a type of knot to establish a mesh, which then forms temporal “lines of becoming” (Ingold, 2007, 2013) involving various types of semiotic resources.

Japanese society is often described as a well-organized entity, structured with a variety of traditional social norms that can be referenced in numerous spheres of social life. However, actual practices may not coincide with social norms. Where this occurs, phrases including *hazukashii* can fill the gap between practice and norm. Then, the actor whose action is regarded as *hazukashii* performs a new action, which can justify, repair, or elaborate a prior action in a contextually appropriate manner. Through such exchanges, speaker and audience can cooperate in establishing an affective stance by which to affiliate with the socially shared normativity (cf. Goodwin et al., 2012; Cekaite and Björk-Willén, 2018). This affective stance is a powerful tool for the language socialization of children in a given speech community. As such, culture is incrementally attained through “the interactively organized process of public recognition of meaningful events” (Goodwin, 2000, p. 1492). In this sense, the caregiver’s communicative style is an important factor in the socialization of children to culture-specific values (Clancy, 1986, p. 218), and discursive practices in caregiver–child interaction construct a culturally distinct self (Demuth, 2013).

In a nutshell, emotional expressions and emotional experiences build sociality, and social activities construct emotions. These two mechanisms do not contradict one another. Rather, recursive interplay emerges through them. By combining the analysis of situated social interaction with ethnographic procedures we can reveal relationships between the two mechanisms to cultivate an interactional study of emotion, building a foundation for the better understanding of our lived culture.

## Ethics Statement

This study was carried out in accordance with the recommendations of the Ethics Review Committee at the Center for African Area Studies, Kyoto University with written informed consent from all subjects. All subjects gave written informed consent in accordance with the Declaration of Helsinki. The protocol was approved by the Ethics Review Committee at the Center for African Area Studies, Kyoto University.

## Author Contributions

AT made major contributions to the design of the manuscript, data collection, analysis and interpretation of data, and drafting the manuscript.

## Conflict of Interest Statement

The author declares that the research was conducted in the absence of any commercial or financial relationships that could be construed as a potential conflict of interest.

## Footnotes

1 Ekman (1992) listed *anger*, *disgust*, *fear*, *happiness*, *sadness*, *surprise*, *interest*, *awe*, *contempt*, *embarrassment*, *enjoyment*, *excitement*, *guilt*, and *shame* as candidates for the basic emotions. These were chosen for the basic emotions because they used distinctive and universal signals, such as facial expressions; other primates exhibited similar emotions. These emotions had distinct physiological functions, the event preceding the emotion showed distinct and universal features, they caused consistent emotional responses, they occurred quickly and they did not last long, they were assessed automatically and they occurred spontaneously.

2 For more comprehensive reviews of this topic, see Sakuta (1967) and Minami (1994).

3 In *The Japanese Journal of Ethnology (Minzokugaku Kenkyu)*, a representative academic publication on Japanese ethnology and cultural anthropology, published reviews of *The Chrysanthemum and the Sword* in 1950, not long after the war. Leading Japanese intellectuals such as Takeyoshi Kawashima (an expert in the sociology of the law), Hiroshi Minami (social psychology), Kizaemon Ariga (rural sociology), Tetsuro Watsuji (philosophy), and Kunio Yanagita (folklore studies) contributed articles to the issue. Although the judgments of Kawashima, Minami, Ariga, and Yanagita were positive overall, they also criticized the work. In brief: they asserted that the Japanese were over-generalized in Benedict’s work; differences owing to social class, geography, occupation, and other factors were overlooked (Ariga, 1950; Kawashima, 1950); the author considered Japanese culture to be a fixed entity; insufficient analysis of the internal dynamics of the culture had been done (Minami, 1950); it would be impossible to improve one’s understanding by contrasting the culture of sin against the culture of shame within one horizon (Yanagita, 1950); and *The Chrysanthemum and the Sword* improperly produced general conclusions from error-ridden or misunderstood data (Watsuji, 1950). All of these criticisms more or less pointed to the limitations of Benedict’s argument and were repeated afterward by others.

4 Along the lines of Doi’s (1973, 1974) argument, Takada (2013) demonstrated that the use of particular grammatical items, such as modal markers, in Japanese conversation make it possible to modulate the intensity of action. These items usually come at the end of a sentence and allow the speaker to coordinate his or her actions with a hearer’s behavior, while progressively monitoring the latter. Additionally, Takada and Kawashima (in press) argued that the dropping of the subject, which often occurs in Japanese conversation, has the effect of blurring the distinctions of footing (Goffman, 1981) among the participants of the interaction. These syntactic features contribute significantly to ambiguate differences in opinion between speaker and hearer, thereby enhancing their empathetic cooperation, one of the important features of *amae*.

5 http://www.cci.jambo.africa.kyoto-u.ac.jp/en/

6 http://www.bun.kyoto-u.ac.jp/sitakura/infant_scientist.html

7 I note here the generous work of Dr. Yoshihiko Asao (National Institute of Information and Communications Technology) in building the search system.

8 In the excerpts, utterances are transcribed according to a modified version of the conventions developed in conversation analysis research (for details, see Schegloff, 2007; Sidnell and Stivers, 2013). Information that is significant for the utterance is indicated in double parentheses: (()). Equals signs (=) indicate run-on utterances or an utterance that has been interrupted by someone else. Pause length is marked in parentheses, in tenths of a second [e.g., (0.6)]. A dot in parentheses (.) indicates a tiny gap, probably no more than one-tenth of a second. Overlap of utterances is marked by square brackets: []. Two degree signs (^∘∘^) enclose remarks that were markedly softer in tone than the discussion surrounding it. Two number signs (# #) enclose indicates a rasping or ‘creaky’ voice quality. An up arrow (↑) marks an increase in the pitch of the voice. Talk between “more-than” and “less-than” symbols has been compressed (> <) or slowed down (< >). A dash after a word or part of a word indicates a cutoff or self-interruption. Period indicates a full stop (pronounced in a falling tone), while comma indicates more is expected (pronounced in a slight falling tone). Colons (:) indicate prolongation of the immediately prior sound and the length of the row of colons indicates the length of the prolongation. A row of h’s prefixed by a dot indicates an inbreath, without a dot an outbreath. The length of the row of h’s indicates the length of the in- or outbreath. Capitals indicate especially loud sounds relative to the surrounding talk. Single parentheses indicate that an utterance was unintelligible or made by an unidentifiable source.

9 Interlinear gloss abbreviations are indicated as follows: ACC, accusative; ASP, aspect marker; CAU, causative suffix; COND, conditional form; COP, copula; CP, conjunctive particle; DAT, dative; DIM, diminutive marker; HON, honorific marker; IJ, interjection; LK, linker; NAME, proper name; NEG, negative; NOM, nominative; PER, perfect; PP, pragmatic particle; PST, past; Q, question marker; QT, quotative particle; SSW, sound-symbolic word; TE, conjunctive (-te form); VOL, volitional suffix.

10 Following the convention of interaction analysis, I describe the interactions in the transcriptions in the present tense to focus on the ongoing nature of the construction of social reality.

11 It is possible to interpret the meaning of this part of the utterance as either “((you should feel)) ashamed” (because she is no longer a baby) or “((mother is feeling)) embarrassed” (because of her child’s babyish behavior in this video-recorded situation). I assume that the former is accurate, because it does not mark a change in the sentence subject either literally (manifesting the sentence subject) or prosodically (e.g., modifying the tone of the voice) from the preceding part of the utterance [“wow(h), ((you)) behaved(h) like(h) a(h) baby(h)”]. Note that it is not obligatory in Japanese to mark the semantic subject and/or actor of a verb when it may be inferred from the semantic or pragmatic context (e.g., Kobayashi, 2005, 2006). Additionally, the fact that both interpretations are plausible shows that the characteristics of *amae* are being socialized here (i.e., the mother’s feeling can coincide with what the girl should be feeling, thus rendering the emotion co-dependent).
